# Teachers and Parents’ Perceptions of Care for Students with Type 1 Diabetes Mellitus and Their Needs in the School Setting

**DOI:** 10.3390/children9020143

**Published:** 2022-01-23

**Authors:** Laura Armas Junco, María Fernández-Hawrylak

**Affiliations:** Department of Educational Sciences, Universidad de Burgos, 09001 Burgos, Spain; larmas@ubu.es

**Keywords:** care, Diabetes Mellitus, Diabetes Mellitus Type 1, illness, nursing, family, hyperglycaemic, insulin

## Abstract

The high incidence of Type 1 Diabetes Mellitus (DM1) increases the likelihood of teachers having students with this illness in their classrooms. The objective of this study is to investigate the needs of students with DM1 during the school day from the perspective of both teachers and parents. A mixed methods study was designed and a questionnaire was administered to practicing teachers in Pre-primary Education, Primary Education, Compulsory Secondary Education, and Further Education, as well as Vocational Education within both the province and the city of Burgos (Castile and Leon, Spain) who may have students with DM1. Semi-structured interviews were also conducted with mothers and fathers, members of the Burgos Diabetics Association (ASDIBUR). In the questionnaires administered to the teaching staff, 54.8% affirmed that they knew of students with DM1 at their centers. Of those questioned, 51.2% affirmed that they knew of the existence of action protocols on DM, and 45.2% declared that they had received specialized information on the illness; 92.8% believed that there was no discrimination at their center towards students with DM, and 82.8% thought that the educational center raised no objections to students with DM departing on trips during the school year. In their interviews, both family and teachers assessed the material and human resources as insufficient and called for the presence of school nurses at the educational centers. It is important to raise the awareness of the educational community about the needs of students with DM1 and to provide guidelines on emergency situations to teachers and staff at the centers.

## 1. Introduction

Type 1 Diabetes Mellitus (DM1), diabetes is a chronic degenerative metabolic illness, due to the destruction of the insulin-producing beta cells [1,2]. The majority of adults, children, and adolescents with DM1 should be treated with daily insulin injections or with insulin pumps. Around one third are diagnosed as diabetic ketoacidosis. Type 2 Diabetes Mellitus (DM2), with its onset in early adulthood, is characterized by a progressive loss of β-cell insulin secretion frequently on the background of insulin resistance. The onset of the illness is gradual and the symptoms, although similar to those of DM1 are usually less intense. It affects 90–95% of people suffering from DM [3].

DM impacts different spheres of life of the patient and is associated with physical, psychological, and social alterations [4,5]. The reactions to DM depend on various factors. Children often experience feelings of isolation, negation, depression, guilt, anger, frustration, resentment, fear, anxiety, shame, and dependence. These emotions can appear when they become aware that they are suffering from DM [6].

Previous epidemiological studies suggested that up to 1 in 4 children have a chronic disease, with prevalence estimates ranging from 10% to 30%, a heterogeneity mainly due to the absence of standardized criteria for the definition of pediatric chronic disease [7]. In 2017, in Spain, there were 1,313,400 children between 0 and 14 years old diagnosed as having some chronic illness, in other words, 19% of the child population up to 14 years old has been diagnosed with some sort of chronic illness [8]. The students enrolled in non-university education in the academic year 19/20 were 8,276,528 in Spain (17.49% of the total population) and 54,117 (15.21%) in the province of Burgos [9,10].

Approximately 10% to 15% of students at school present chronic health pathologies, but DM is one of the most frequent, with a high incidence of new cases among students each year [11,12]. The average incidence of DM1 in Spain is approximately 17.7 for each 100,000 inhabitants under 14 years old, fluctuating between 7.9 reported in the Balearic Islands and 30–36 in the Canary Islands. Approximate data for the Autonomous Region of Castile and Leon is 16.7–18.8 [12]. At this age, patients normally present type 1 and an estimated 1200 new cases are reported each year among children under 14 years old, affecting some 15,500 children and adolescents (under 19 years old) [13]. Precisely due to this high incidence, it is highly likely that the teachers, throughout their long educational practice, will have had a student with DM1 in their classrooms [14,15].

There are neither many historical nor recent studies on the inclusion of students with DM in Spain, nor in the provinces. Between 2004–2005, the Fundación para la Diabetes (FD) conducted a descriptive study in the Community of Madrid with parents of children with DM of school age (3–18 years), with the purpose of detecting the real needs of these young people in their school life and to know the difficulties for their complete integration in daily life [16]. Some results were: (a) parental demand for a nurse at each educational center and better-informed teachers with regard to DM; (b) something less than half of the parents had to change their working arrangements to attend to their child; 30% of students ate at school, of whom a broad segment affirmed that food was not controlled; (c) in the opinion of the parents, only 35% of Physical Education teachers knew how to recognize the symptoms of hypoglycemia; (d) the children never went on outings and out-of-school trips overnight, despite the absence of any objection from the school. Some years after they obtained these results, the FD [17] conducted a new study in 2014–2015 to survey the situation at that time of the children of school age not only within the Community of Madrid, but throughout Spain, as well as the demands of parents and, of course, the children. Among the results, we may highlight that: (a) parents called for greater general information for teachers on emergency situations in the classroom and in common rooms, and a nurse at the educational center; (b) changes to the working arrangements of parents; (c) the students carried out Physical Education activities in the same way as their companions; (d) no use of the school dining room to ensure that the students follow a good diet and the inject insulin in a proper way; (c) parents declared that the centers placed obstacles to their children going on school trips. Other research works from the point of view of the teaching staff [11,18] highlighted that a high percentage of teachers have had students with DM in their classrooms, that they would not know what to do in (hyper or hypoglycemic) emergencies, and that the educational centers are not completely equipped to care for these students. Although the situation has improved in some ways, such as in sports practice at school, not all the necessary measures are adopted to contribute to the integration of diabetic students at school, ensuring their rights as chronic patients. Parents are continuously calling for more information for teachers and for staff at educational centers, and the appointment of a health worker at each educational center [19].

DM-related health costs have turned into one of the main public health problems due to their high associated human and economic costs. Moreover, both the economic and the intangible costs, as well as emotional problems, have a great impact on the lives of children diagnosed with DM and their families [13,20], which therefore makes it necessary to extend this knowledge in matters concerning chronic illnesses, such as DM, to the training of educational practitioners.

The quantitative component of this mixed methods study emerged from the following research question: What is the knowledge and what are the attitudes of teachers and parents toward care for students with DM1 in the school setting? The qualitative component was centered on two key questions: (1) How do parents and teachers describe the care services at the educational center for students with DM1 during the school day?; (2) In what way are the educational and care needs of the students with DM1 being covered?

## 2. Materials and Methods

### 2.1. Research Objectives and Design

The general objective of this study is to know the needs of students with DM1 during their school day from the perspective of both teachers and parents. The specific objectives are: (1) To determine the level of awareness and the attitudes of teachers with regard to DM; (2) To describe the care at educational centers for students with DM.

An explanatory, sequential, mixed methods, research design was prepared [21,22,23]: a non-experimental, observational, descriptive, transversal, and retrospective quantitative design [21,24]. The information gathered was complemented with a qualitative phenomenological design [25]. The motivation to use a mixed methods approach was in recognition of the limits on delving deeply into knowledge of the needs of students with DM in the school environment, solely on the basis of data from questionnaires without considering their qualitative nature [21]. This two-phase design started with the collection and analysis of the questionnaire data (quantitative data), followed by the collection and the analysis of information from interviews (qualitative data). All the quantitative and qualitative aspects were equally weighted.

### 2.2. Participants

The sample consisted of 652 practicing teachers from Pre-primary Education (IE), Primary Education (PE), Compulsory Secondary Education (CSE), Further Education (FE) and Vocational Training (VT) at education centers within the province and the city of Burgos from a representative sample of the population [10] and 15 parents (members) of ASDIBUR (Asociación de Diabéticos de Burgos).

### 2.3. Instruments

The study was developed in the academic years 2018/2019 to 2020/2021. Data collection in the quantitative phase took place throughout these first two academic years. A questionnaire specifically designed for the investigation was administered to teaching staff (adaptation of a questionnaire on quality of life prepared at the Endocrinology and Nutrition Service of the University Hospital of Burgos, and other study instruments of the FD), formed of both socio-demographic questions and others aimed at knowing the implications of DM at school from the perspective of the teachers. The aim, with this instrument, was to collect information on attention to educational and care needs of students with this illness, and to pick up any relevant concerns of the teaching staff. The questionnaire consisted of 39 open, closed, or combined questions. It included an Inventory of Negative Attitudes toward Students with DM1 (INAAD) with a 1–5 point Likert-type scale of 25 items. The questionnaires were sent out and personally collected at the educational centers, prior to a request to the governing body together with written informed consent of participants. They were administered to the teachers at the educational centers in a voluntary manner. Cronbach’s α was applied at various points of the Inventory, to evaluate the degree of reliability and validity of the database used in this study, which meant those items that lowered the reliability coefficient had to be removed, finally arriving at a value of 0.718 that was interpreted, although rather cautiously, as significant.

Semi-structured interviews were also carried out individually with partners from ASDIBUR (parents) and practicing teachers who had had students with DM1 in their classrooms, directed at understanding the perspectives of the respondents in their daily lives with the situation under study, in other words, their perspectives as mothers, fathers and teachers of children with diabetes. The interviews were carried out and transcribed by an expert during the last academic year. Participation was voluntary following the ethical criteria of confidentiality and informed consent, recording and purpose of the results. The questions were directed at knowing their perceptions on the care provided to children with DM at the educational centers.

### 2.4. Ethical Considerations

A favorable report was received from the Bioethics Committee of the University of Burgos, complying with ethical norms and, in particular, with the contents of the Helsinki Declaration [26] for research involving human beings. It also complies with the provisions of the General Data Protection Regulation (EU) (GDPR) (2016), Constitutional Act 3/2018, of 5 December, on the Protection of Personal Data and Guaranteeing Digital Rights, and Act 14/2011, of 1 June, on Science, Technology and Innovation [27,28,29].

### 2.5. Data Analysis

The data collected in the quantitative phase were analyzed first, which provided the basis for the qualitative phase [30].

#### 2.5.1. Quantitative Phase

With the data from the questionnaires, a research base was generated in the statistical software SPSS (version 25.0) licensed to the University of Burgos. A statistical analysis was completed first through the application of frequency tables for the variables that were considered for evaluation. A histogram was performed with all the variables comprised in the Inventario de Actitudes Negativas hacia la Atención del Alumnado con DM1 (INAAD, Inventory of Negative Attitudes toward Caring for Students with DM1). A histogram was prepared with all the variables from the 1–5 point Likert-type scale, from which an in-depth analysis was performed of the responses of the teachers. Subsequently, two tables showing the comparison of means were prepared. Statistical inference tests were likewise applied to determine conclusions that might be extrapolatable and generalizable in other population samples. The non-parametric test of the independence of the variables, Chi-squared (X^2^), followed by parametric tests such as the Pearson correlation were applied. The non-parametric Mann–Whitney U-test comparison of means test was also performed. Finally, a contingency table was prepared with the subsample of variables (IE/PE and CSE/FE/VT) and the question “have you on some occasions helped your students to control their glycaemia during the school day?”.

#### 2.5.2. Qualitative Phase

The interviews were transcribed in full and various in-depth readings were performed. A categorical-thematic analysis was carried out, considering the presence and the absence of terms or concepts that are independent of each other, and inductive, through the categories that emerged from the content [31,32]. It meant that categories with topics that were widely related to our themes could be identified in longer sections of text with our qualitative research questions [33]. As part of this process, the specific parts related to pertinent topics are highlighted, as are the most clearly articulated and relevant citations that illustrated a particular topic for inclusion in the analysis. The organization of the results was done by researchers paying attention to the relevance and the frequency of the citations, and they then interpreted the data following the established methodological lines of analysis.

#### 2.5.3. Integration and Interpretation

We implemented the integration of the data through structural connections. We invited several teachers to participate in the interviews. They were very aware of DM1 and had previously expressed their interest in the study and their willingness to collaborate during the quantitative phase. Likewise, a selection of parents of children with DM1, some of whom had informed ADISBUR that they had experienced discrimination at school. This information meant that the perceptions of all the participants could be juxtaposed. The fundamental dimension of interest was perception of the discrimination of students with diabetes, which was determined by comparing the data from the questionnaires with the narratives from the interviews. A small percentage of teachers, according to the quantitative results, suggested discrimination, and a slightly larger percentage suggested the incapability to respond to emergency situations. We used their responses to develop the script of the interviews to investigate these percentages. The implementation of integration at the level of interpretation and reporting was through the narrative texts.

## 3. Results

Consistent with the design of quantitative and qualitative mixed methods design, we prepared quantitative and qualitative research questions in sequential order, to achieve a broader and deeper perspective with which to approach DM1 in the school environment. Remaining faithful to this design, which is of vital importance for the validity of the investigative methods [34] we will present the results of each one separately before their integration and interpretation in the discussion.

### 3.1. Results of the Questionnaires

A total of 652 questionnaires were administered to teachers (439 women and 212 men) at 108 educational centers (73 public and 35 state-aided). With regard to the place, 66 of the centers were found in Burgos capital and 42 in the province with the same name. The ages of the group of 606 participants who filled in the questionnaire varied between a minimum of 23 up to a maximum of 65 (A = 43.48, SD = 9.66). The highest number of teachers (191) was situated in the age range of 41 to 50 years old. It was observed that 325 participants were tutors and 324 directors, heads of study or others. The number of years the teachers had given class were as follows: 129 teachers (0 to 6 years old); 123 (7 to 12 years old); 129 (13 to 18 years old); 102 (9 to 24 years old); 86 (25 to 30 years old), 47 (31 to 36 years old) and 17 (37 years old or over). With regard to the educational stage, 347 teachers were teaching Pre-primary and Primary Education and 305 teachers were teaching Compulsory Secondary and Further Education, and Vocational Training (Table 1).

In all, 54.8% of teaching staff (a total of 357 of 652) affirmed that they knew of students with DM1 at their educational center. 46.9% (306 of 652) were teachers of students with DM1. 18.7% were tutors of students DM (122 of 652). Of teaching staff, 51.2% (334 of 652) affirmed that they were aware of the existence of action protocols on DM and 45.2% (295 of 652) said that they had received specialized information on the illness. In relation to the teaching group, 12.3% (80 of 652) had received some type of course on DM at some point in their life. In all, 92.8% (605 of 652) believed that there had been no discrimination toward students with DM at their educational center. Furthermore, 82.8% (540 of 652) considered that the educational center placed no barriers on students with DM from going on school trips of two days.

**Figure 1:**
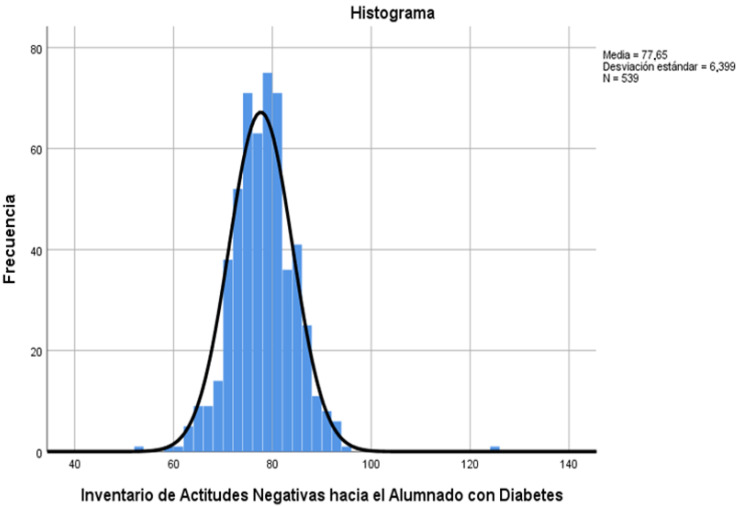
Global analysis of the scale.

With regard to the global analysis of the scale, Figure 1 shows the average score that the teachers obtained, with a minimum score of 53 and a maximum of 125. The highest scores were considered to be negative attitudes among the teachers toward the illness and the lowest pointed to positive attitudes toward DM1 and its normalization in the classroom. The average score was 77.65 (SD = 6.399), which indicated a positive tendency toward the illness and the educational attention that it receives.

The scores obtained were placed on the scale with other variables from the questionnaire such as having been a teacher of students with DM1 or having received specialized information on the illness. After conducting the Student-t test of the difference of means, it was observed that the level of significance with a value of *p* = 0.845 > 0.05, verified the presence of homogeneity between having had or not having had students with DM1 in relation to the Inventory (Table 2).

Another Student-t test of the difference in means was conducted to see whether having received specialized information or otherwise on DM1 improved the attitudes of the teachers. A *p*-value = 0.760 > 0.05 was observed, which pointed to the presence of homogeneity between the teachers who had and who had not received specialized information in relation to INAAD (Table 3).

The Kolmogorov–Smirnov test of the normality of means confirmed a significance value of *p* < *0*.05 and the Chi-squared test was applied to the variables (and Action Protocol), which yielded distributed results with a value of *p* = *0*.258 > 0.05. Results that mean we cannot reject the null hypothesis, therefore accepting that the type of educational center had no influence on its relation to the existence of a diabetes action protocol. The Chi-squared test yielded a significance level of *p* = 0.001 < 0.05, hence the existence of a relation of dependency or influence between the type of center and the appropriateness of the equipment at the educational center. A Pearson correlation test applied between the two variables, tutor and teacher of students with DM1, presented a correlation of r = 0.486, distributed with a value *p* = 0.000 < 0.05, and a positive correlation for social contact between the students with DM1 and the teacher/tutor. The teachers who were also tutors of students with DM1 showed greater affinity than those who were only either a tutor or a teacher. A significative relation was also found for social contact between students with DM1 and the teacher/tutor. A significative relation was also found between being a teacher of students with DM1 and awareness of the existence of an action protocol on Diabetes, highlighting a value of r = 0.424 and a value of *p* = 0.000 < 0.05. The Mann–Whitney U-test was applied after having completed the corresponding Kolmogorov–Smirnov test, which yielded a lower significance level than 0.05. This test yielded a distributed result with a value of *p* = 0.100 > 0.05, data that led us to conclude that a teacher, regardless of sex, adequately cares for students with DM1 in the same way. Finally, a contingency table was prepared where it was observed that in the educational stage corresponding to compulsory secondary and further education and vocational training, 87.5% of teachers helped students with DM1 to perform their glycemia tests during the school.

### 3.2. Results of the Interviews with the Teachers (n = 11) and the Families (n = 15)

Qualitative interviews were individually conducted with 11 teachers from Pre-primary and Primary Education, Compulsory Secondary and Further Education and Vocational Training, four women and seven men, from public sector and state-aided educational centers within the province of Burgos, who have had students with diabetes in their classrooms, with the exception of two, and who had between 3 and 22 years of teaching experience; and 10 mothers and 5 fathers of children with DM1, between 5 and 18 years old, attending classes at these educational centers in all levels of education.

A total of seven categories were established in the interviews with the teachers and eight in the interviews with family members.

The categories for the interviews with teachers were as follows:

*Academic Performance*

Almost half of the teachers (5) were of the opinion that DM1 affected the academic performance of the students. One teacher said: “*Yes, it affects [the student] in the sense that the time spent outside the center means, well, that the student loses the habitual pace of the classes. (…) It may demotivate them*” (Teacher 1).

*Attention to Students with DM*

With regard to the attention toward students with DM1 from the center, almost all the teachers (9) agreed that it was good. One teacher put it as follows: “*I think that the centers ensure that both boys and girls receive adequate attention (…) In cases where there is a diabetic at the center, then they take steps to ensure they have the necessary resources*” (Teacher 11).

*Measures That Are Taken or Might Be Taken in the Classroom to Respond to the Needs of Students with DM1*

All the teachers were in agreement that they would take the following measures in the classroom to cover the needs of student with DM1: follow the guidance given by the families, orientation and advice, as well as presenting more tutorials and informative talks on the illness. One teacher highlighted the importance of the close contact with the families: “*Once we are told that there is a case, the first thing is of course to talk with the family, to see how they are coping with the process and, well, encourage them in every way we can. They can rely on me for what they need and when speaking with the management team*” (Teacher 10).

*Attitude and Behavior of Classroom Companions*

All the teachers agreed that the attitude and the behavior of their classmates had been good. One teacher said: *“I think that diabetes is a task. It’s an illness that if it is really well monitored at a physical and at an emotional level does not have to impact on the child in relations with other classmates, because in the end, it’s something that… when well controlled, day to day activities can be developed and there’s no reason for the relations with classmates to be affected”* (Teacher 11).

*Training at the Educational Center to Respond to Emergency Situations*

In reference to training at the educational center to attend to emergency situations, for over half of the students (7), the training and information was insufficient: “*We have received no training. At the centers, at least in those where I’ve been, I’ve never seen training given on diabetes*” (Teacher 11). Some teachers said (3) openly that they would not be prepared to intervene in a diabetic emergency: “*I don’t think so, really, what I would do would be to call 112 and ask, and I’d do whatever they tell me to do, but I honestly don’t think that I am prepared*” (Teacher 6).

*The School Nurse*

The school nurse was well-known by almost all the teachers (8), but they had no nurses at their educational centers. One teacher ironically said: “*I have heard speak of her and we’ve struggled for it to happen, but she’s hasn’t turned up anywhere* (laughs)” (Teacher 7).

*Situation in Which the Students with DM1 Were Not Permitted to Join in the Same Activities as Everybody Else*

Almost half of the teachers (6) considered that there were situations in which the students with DM1 had not been able to carry out the same activities a everybody else. One teacher narrated this situation of discrimination: “*Yes, I have heard of cases of, for example, excursions, well, they never let the student go on the excursion because the teachers were fearful of having to take it in hand, having to inject the insulin, having to give the medication, having to oversee them in case of hypoglycemia. Yes, I have heard of these situations yes, above all in relation to excursions and I have also heard of cases of teachers who refused to help to administer the insulin, because they said that it was not their responsibility*” (Teacher 9).

The categories in the interviews with the families were:

*Academic Performance*

Over half of the families (9) expressed the opinion that DM1 affected the academic performance of their child, producing above all distraction and loss of concentration. One mother described how the (high-low) sugar levels affected her son: “*In many ways. This is a function of the sugar levels he has at the time. If the level is low: he’s distracted, sleeps, doesn’t register much in class, pays little or no attention and if the level is high, also*” (Family 2).

*Attention from the Educational Center*

Attention is provided by the school community, coming principally from the teacher and the management team. Over half of the families (8) had a good perception of the attention received by the center, highlighting their collaboration, involvement, and trust. However, three families thought that the attention was poor, emphasizing the lack of involvement of the center, although all three linked this poor attention with the independence of the children due to their age or to particular situations: “*Right now, in no way. Right now, when they are adolescents, they are independent. They’re the ones who solve it all*” (Family 10). A mother expressed her discontent with the school trips: “*Yes, I have had problems, for example, with the school trips*” (Family 3). This mother described a situation in which she had to defend the right of her daughter to go on the trip and to be cared for so that her illness might not be seen as a handicap.

*Measures Established by the Center*

Over half of the families (9) were satisfied with the facilities, thanks to the measures established by the center, but in the end, attention was dependent on the willingness of the teachers: “*In the institute there was a Physical Education teacher who told her that if she had any need for glucagon then she should give it to herself*” (Family 2). Somewhat less than half of the families (6) expressed their unease with how their children had been treated, because they considered that the center should have involved itself more. This was what one father said: “*It has solely and exclusively been resolved among us* (laughs)” (Family 13). They described how on occasions the lack of adequate attention at the center was to their detriment, as they had to travel to the center to control the glucose levels of their child: “*I stopped working (…) It was later on just when I started to be a little more independent, I have returned to work, but to hold on to a job…*” (Parent 2). “*I don’t exactly work for diabetes, because since I started there was no way that they could manage*” (Parent 10). “*There are a large number of parents who have to stop work to go and give the glycemia to the kid, because there were teachers who whether right or wrong, let’s not get into that, well they refused, because they obviously did not have that training and were scared*” (Parent 14).

Others exempted the teachers from any responsibility (6) to avoid legal impacts. As an example, the comments from this father: “*We have also requested, we have always signed documents asking for collaboration, exempting the teacher from any responsibility so that there’s no pressure*” (Family 7).

*Attitude and Behavior of the People in the School Environment*

Practically all the families (14) considered that the attitude and the behavior among classmates toward their children were good. Many families (10) wished likewise to highlight the kindness and involvement of the teachers. One father made special emphasis of the normalization of the illness: “*The teachers treat him as another child. There is no special treatment. They are simply aware of the situation. Aware that the child has the illness and that there is some attention, but well, like for other children who might have some other types of pathology*” (Family 8).

*Training of the Educational Center to Deal with Emergency Situations*

The center, in the opinion of somewhat over half of the families (8), were without the necessary human and material resources to respond to emergency situations. One mother came out with the following explanation for the lack of resources: “*I don’t think it’s because they are overstretched (…) They don’t have enough (…) today no*” (Family 15). The training and information were insufficient for most of the families (12). One mother complained: “*Well, it’s not enough nor is it sufficient, I think that they should, well they should push a little bit more, to explain a little what diabetes is to train teachers because they are not all aware of it*” (Family 3).

*The Role of the School Nurse*

Most of the families (12) knew of the role of the school nurse, but had no nurse at their children’s educational center. One mother said in an ironic tone: “(laughs) *I don’t know her because she doesn’t exist, but I know what the role is*” (Family 4). The school nurse would bring peace of mind, relief, and help for all the families. One father commented: “*Well we’d all be calmer, I think that above all she would bring peace of mind and to know that any doubt that he has, that nurse will solve it and will help him and will guide him*” (Family 7). A little less than half of the families (6) called for the presence of the school nurse. For example, one parent spoke of the need for coordination to be able to introduce this role at the centers: “*(…) What I do think would be desirable might be coordination between education and health to introduce the role of the health worker to the schools. It would be desirable for all the parents*” (Family 9).

*Situations of Discrimination*

All the families, except for one, agreed that their children had suffered no discrimination. The situations of discrimination occurred on school trips, and one father expressed as much: “*There were excursions and the only way that our child could go on them, could participate in an outing, perhaps eating there, returning in the afternoon and the day had passed by, was because a family member went with the child, because if not, there was no teacher willing to assume responsibility for injecting the insulin or helping the child with the calculation of units that have to be injected… no, in that sense we never had that sort of help*” (Family 7).

*School Dining Room*

Six families considered that nutritional information was unknown among the staff. Some reasons that they mentioned were a lack of preparation and of experience, insecurity and doubts. One mother said: “*Right now, it he had to stay in the dining room, no, I wouldn’t hold out may hand. I’ve never explored it because I haven’t contemplated that possibility, but they are not prepared, no, no, far less so, because the teachers who know him don’t stay in the dining room*” (Family 5).

## 4. Discussion and Conclusions

The objective of this study has been to investigate the needs of students with DM1 from the perspective of both teachers and parents.

Almost all teachers will at some time in their career have a student in their class with a chronic illness. Academically speaking, diabetes in children neither affects their intellectual capacity, nor on their reasoning, nor on their social capabilities, which means that they need no individualized curricular adaptations, but they do need supervision in those activities that require greater physical effort and food intake, with glycaemia tests adjusting the insulin doses and dietary supplements according to individual risk-related circumstances of presenting with symptomatic hypoglycemia [15].

The results were consistent with others that described the implications that various chronic illnesses have at school in the context of Spain [15,35] and others centered on DM1 [11,16,17,18].

In the present study, almost half of the teachers who were interviewed (46.9%) were teachers of students with DM1. A small percentage of teachers (12.3%) had attended some sort of course on DM at some point in their lives. Comparable results were obtained in another similar study completed in Cadiz [18] where it was demonstrated that almost half the teachers who were interviewed (43.2%) had taught students with DM1 and only 0.8% acknowledged having received training on diabetes. In a similar study within the Community of Madrid in 2009 [11], 71.1% of the teachers who completed the survey recognized having had some student with DM1 in their classes, and that the degree of diabetes-related knowledge among teachers was low-to-medium. Similar data was obtained in 2007 [16] where only 34% of the families believed that the teachers could recognize the symptoms of a light hypoglycemic episode; 17% of families had problems at their educational centers, 5% of the children were not accepted and 8% were obliged to change school: in some cases, they had to modify the control of their glucose (9%) and administration of treatment (16%) in view of the lack of cooperation from the school.

In another study on chronic illnesses completed in Galicia [15], 59.8% of teachers had students with chronic illnesses in their classrooms. Their main concern was not knowing how to act in a potentially serious situation. Another recent study completed in Asturias that also focused on chronic illnesses [35] reported that 73.9% of teachers had students with chronic illnesses, that 45.5% had action protocols for accidents and health emergencies and that only 14.9% had received some type of specific training on students with chronic illnesses.

In our study, the teachers considered that lack of information and training were the principal obstacle to providing attention to students with DM. Even so, many of them— 58.4% of teachers (381 of 652)—declared that it would be necessary to contract specialized health personnel to assist students suffering some sort of health problem, calling moreover for the presence of a school nurse at the centers, with a special emphasis since COVID-19, among parents and teachers. The parents interviewed in the present study pointed out that a school nurse would be good for *“any type of illness and to respond to emergency situations”*. One parent said: *“Sure, I think that it appeared important to me but not only for one thing, evidently diabetes is my area, but I can imagine other problems for any other type of child. It is a way of directly approaching that without the parents having any sort of problem and in some way you have peace of mind. I think that the nurse is a very important role, which is not well, because… like everything, there won’t be any money, or there won’t be that enthusiasm, but I think that it would be quite important above all in this illness”* (Family 14).

Similar results were found in a nationwide study completed in 2014–2015 by the Community of Madrid where the parents demanded the presence of a nurse and more information for teachers and staff at the educational center [17]. A study completed in Asturias also yielded quite similar results where 51.5% of the teachers surveyed proposed the incorporation of the school nurse as a priority option to improve assistance in case of emergencies [35]. This is important, because there can be factors such as geographic distance from home to the place of work that can prevent parents from immediately attending the school to test the glycaemia levels and administer the insulin injection and the teachers can also be fearful of the responsibility that it entails, as detected in this study.

In another recent study, the authors concluded that the health care of students with chronic diseases in schools can be improved for teachers, pediatricians and pediatric nurses, considering the figure of school nurse as the main improvement measure [35]. In Spain, school nursing is not regulated in national legislation, so it is not present in all Spanish regions or in all types of schools. It is currently recognized in Madrid, Catalonia, and the Canary Islands [36]. This means that there is a clear inequality between the autonomous communities, where the school nurse is more present in the Community of Madrid than in other regions. The Ministry of Education of Castile and León hired 27 nurses. In the public schools of this community, nursing is regulated only in special education centers and occasionally in educational centers that have students with a disability or with a chronic disease [37].

In all, 98% considered it appropriate for teachers to have the symptoms and the steps to follow in the case of hypoglycemia in writing when the student enters the school [11]. In our study, 53.3% of teachers were concerned over the legal responsibility involved in attending to students with DM1 in the classroom, in contrast with 22.6% who presented greater indecision, 32.0% doubted their capability to attend to students with DM1 and 39.1% showed themselves undecided over their capabilities to act in the case of emergency. Some autonomous communities have been designing regulations to attend to students with illness, as in the autonomous community of Castile and Leon [38,39]. The availability of adequate resources, teacher training, and the establishment of action protocols with students with health problems are fundamental. Given the improvements developed over recent years in the treatment protocols to manage different illnesses, many of the experiences of the children have changed for the better. Specifically, the protocols designed in the Autonomous Region of Castile and Leon could have contributed to 92% of the teachers in our study considering that there had been no discrimination toward students with DM at their educational center and that 82.8% held the opinion that the educational center raised no obstacles to students with DM going on school trips during the school year.

Together, the data from the questionnaires and the interviews unearthed the perceptions of the teachers and parents on the needs and the attention lent to students with DM1 in the school context. However, in the light of the questionnaire scores, with respect to what was said in the interviews, the participants can have well-based beliefs on the attention provided to students with DM that are less acceptable in an individualized, critical and in-depth analysis than in a broad-focused and social one. In general, the questionnaire data and the interviews indicated that despite the problems that DM may have in the classrooms of Burgos, province and capital, it is not representative of an initial conflict with respect to the integration of these students and discrimination against them. Nevertheless, according to the questionnaire data, practically all of the teachers thought that *“there is no discrimination within the center toward students with diabetes”*, some parents and teachers narrated in the interviews anecdotes of discrimination linked to the school trips over a few days.

These narratives of teachers and families also suggested limitations to attend to students with DM1 who could be linked with staff training and the willingness of teachers to control glucose levels and to administer insulin, especially in the stages of Pre-primary and Primary Education, but also with the burden of responsibilities and legal consequences. 33.2% Pre-primary and Primary Education of teachers had on some occasion helped students to perform some glycemic control during the school day, as against 12.5% of teachers of Compulsory Secondary and Further Education and Vocational Training, who never considered helping the students, because they had a higher degree of autonomy. These data were consistent with the information from the interviews, which suggested that the participants, both parents and teachers, shared similar perceptions, in so far as the students were going to continue to manage the illness independently as they grew older: *“We said to them that is what had to happen at Secondary School, that they were going to be much more independent than at Primary school, because they are older now and, so they won’t be so dependent upon you and the families will no longer have to go to the center as much”* (Teacher 10); *“I hope that I have changed, above all for the Primary school children, because they then become more independent”* (Parent 2).

DM1, despite being one with the highest prevalence in comparison with other pre-primary chronic illnesses, has some aspects in common with them. The study could be of used to help policy makers to develop regional policy, including coordination protocols between the health and the educational system, to increase opportunities, to improve the quality of life of these children, and to establish interventions focused on inclusive and healthy schooling. It also appears necessary to compile different registers by province and by illness to detect shortcomings in the attention that is provided. It is worth highlighting, in the context of this work, the need to incorporate content related to chronic illnesses and of long duration at school.

Obtaining a representative sample in the quantitative phase, together with the qualitative approach to examine the phenomenon in depth, have made it possible to contribute a meticulous description of the present situation for students with DM1 in the province of Burgos, which constitutes a strong point of this study. The design of a mixed method highlighted the discrepancies that can arise from social desirability and stress when sharing personal points of view and family and professional experiences on daily attention to children with DM in the school setting.

This study presents some limitations. On the one hand, there is an absence of state-sponsored studies on the inclusion of students with chronic illnesses by communities and specific studies on the chronic illnesses of children in the context of schooling in Burgos capital and province, to compare the results. On the other hand, there is a saturation of teaching staff being asked to fill in questionnaires for educational studies organized by universities. This notable fact is turning the completion of questionnaires into a demotivating experience, so much so that the respondents either do not answer all the items or do so without rigor. In particular, were the teachers in Compulsory Secondary and Further Education and Vocational Training, who, due to the burden of schoolwork arising from the pandemic, were very reticent over their completion. It would have been appropriate to interview parents of children with diabetes who do not belong to ADISBUR, but it was not possible. Schools do not provide data to people outside the educational community due to the Data Protection Law (GDPR, 2016). This could be considered a bias as these parents may be more involved in the care of the disease and have more knowledge than other parents who do not belong to any specific group. However, these facts could never significatively modify the results of the objective of the study. Studies on the needs of children with chronic illness have been based on information from families and teachers, instead of listening to the voices of the children themselves; a line of study worthy of consideration.

In conclusion, DM, as with many chronic illnesses, cannot be an obstacle to the inclusion of students at an educational center. Whatever the age at the onset of DM, to minimize the impact of the diagnosis, it is important to return to normal life as soon as possible, and the educational center forms part of that daily life in childhood and in adolescence. In this sense, teachers must be equipped with techniques and tools to guarantee a safe and healthy environment, and to assure participation for all students in all school activities. The needs of students with DM1 must be respected in the classroom to help with the physical, social, and emotional adaptation of these students and to have the human and material resources to attend to them at the educational center. Responsibilities and guidelines for the steps that teachers and staff at the center should follow in emergency situations will be another of the aspects to organize.

## Figures and Tables

**Table 1:** Characteristics of the sample.

Variables	Categories	N	Pct
Sex	Men	212	32.5%
	Women	439	67.3%
	No answer	1	0.2%
Age range	20 to 30	64	9.8%
	31 to 40	179	27.5%
	41 to 50	191	29.3%
	51 to 60	153	23.5%
	61 or over	19	2.9%
	No answer	46	7.0%
Function at the center	Tutor	325	49.8%
	No tutor (principal, head of studies, other staff)	324	49.7%
	No answer	3	0.5%
Years spent teaching	0 to 6	129	19.8%
	7 to 12	123	18.9%
	13 to 18	129	19.8%
	19 to 24	102	15.6%
	25 to 30	86	13.2%
	31 to 36	47	7.2%
	37 or over	17	2.6%
	No answer	19	2.9%
Educational phase	Pre-primary and Primary Education	347	53.2%
	Compulsory Secondary and Further Education and Vocational Training	305	46.8%
Type of center	Public schools	73	67.6%
	Publicly funded private schools	35	32.4%
Location	City of Burgos	66	61.1%
	Province	42	38.9%

**Table 2:** INAAD relationship with having been a teacher of students with diabetes.

	Teacher of Students with Diabetes	N	M	SD	t	Gl	Bilateral Sig.
**INAAD**	Yes	268	77.61	6.391	−0.195	534	0.845
	No	268	77.72	6.429			

**Table 3:** INAAD relationship with having received specialized information.

	Specialized Information	N	M	SD	t	Gl	Bilateral Sig.
**INAAD**	Yes	252	77.58	6.409	−0.306	533	0.760
	No	283	77.75	6.413			

## Data Availability

The data presented in this study are available on request from the corresponding author. The data are not publicly available due to privacy reasons.
